# High Mortality among 30-Day Readmission after Stroke: Predictors and Etiologies of Readmission

**DOI:** 10.3389/fneur.2017.00632

**Published:** 2017-12-07

**Authors:** Amre M. Nouh, Lauren McCormick, Janhavi Modak, Gilbert Fortunato, Ilene Staff

**Affiliations:** ^1^Department of Neurology, Hartford Hospital, Hartford, CT, United States; ^2^Research Administration, Hartford Hospital, Hartford, CT, United States

**Keywords:** 30-day readmission, ischemic stroke, hemorrhagic stroke, Transient Ischemic Attack, stroke mortality

## Abstract

**Background:**

Although some risk factors for stroke readmission have been reported, the mortality risk is unclear. We sought to evaluate etiologies and predictors of 30-day readmissions and determine the associated mortality risk.

**Methods:**

This is a retrospective case–control study evaluating 1,544 patients admitted for stroke (hemorrhagic, ischemic, or TIA) from January 2013 to December 2014. Of these, 134 patients readmitted within 30 days were identified as cases; 1,418 other patients, with no readmissions were identified as controls. Patients readmitted for hospice or elective surgery were excluded. An additional 248 patients deceased on index admission were included for only a comparison of mortality rates. Factors explored included socio-demographic characteristics, clinical comorbidities, stroke characteristics, and length of stay. Chi-square test of proportions and multivariable logistic regression were used to identify independent predictors of 30-day stroke readmissions. Mortality rates were compared for index admission and readmission and among readmission diagnoses.

**Results:**

Among the 1,544 patients in the main analysis, 67% of index stroke admissions were ischemic, 22% hemorrhagic, and 11% TIA. The 30-day readmission rate was 8.7%. The most common etiologies for readmission were infection (30%), recurrent stroke and TIA (20%), and cardiac complications (14%). Significantly higher proportion of those readmitted for recurrent strokes and TIAs presented within the first week (*p* = 0.039) and had a shorter index admission length of stay (*p* = 0.027). Risk factors for 30-day readmission included age >75 (*p* = 0.02), living in a facility prior to index stroke (*p* = 0.01), history of prior stroke (*p* = 0.03), diabetes (*p* = 0.03), chronic heart failure (*p* ≤ 0.001), atrial fibrillation (*p* = 0.03), index admission to non-neurology service (*p* < 0.01), and discharge to other than home (*p* < 0.01). On multivariate analysis, index admission to a non-neurology service was an independent predictor of 30-day readmission (*p* ≤ 0.01). The mortality after a within 30-day readmission after stroke was higher than index admission (36.6 vs. 13.8% *p* ≤ 0.001) (OR 3.6 95% CI 2.5–5.3). Among those readmitted, mortality was significantly higher for those admitted for a recurrent stroke (*p* = 0.006).

**Conclusion:**

Approximately one-third of 30-day readmissions were infection related and one-fifth returned with recurrent stroke or TIA. Index admission to non-neurology service was an independent risk factor of 30-day readmissions. The mortality rate for 30-day readmission after stroke is more than 2.5 times greater than index admissions and highest among those readmitted for recurrent stroke. Identifying high-risk patients for readmission, ensuring appropriate level of service, and early outpatient follow-up may help reduce 30-day readmission and the high associated risk of mortality.

## Introduction

Stroke is a prevalent, costly, and detrimental disease. According to the American Heart Association, stroke is the fifth leading cause of death and the second leading cause of disability. In the United States alone, it is estimated that 795,000 people suffer new or recurrent strokes each year, totaling roughly $65 billion in annual direct and indirect costs (1). The mean lifetime cost of ischemic stroke is $140,048, placing stroke among the top 10 most costly conditions among Medicare beneficiaries (2). This disease burden is twofold including initial hospitalization costs and subsequent costs of readmission due to stroke-associated deficits often resulting in continuous risk for hospital readmission.

Previous studies have estimated that as many as 21% of stroke patients are readmitted within 30 days and greater than 55% are readmitted by 1 year (3). Recent studies have found that unplanned Medicare readmission in 2004 estimated in excess of $17 billion in costs (4). Reducing readmission rates among hospitals has become a goal of national healthcare reform and the Centers for Medicare and Medicaid Services (CMS). CMS has defined 30-day readmissions as indicative of poor hospital inpatient care, and therefore links it to payment determination and penalties for hospitals (5).

To reduce 30-day readmission after stroke and mortality, it is imperative to understand preventable and unpreventable predictive factors that may influence readmission. This study had two basic aims: to explore risk factors that lead to 30-day readmission following a stroke and characteristics of the readmission stay, including in-hospital mortality. Risk standardized models are available for conditions such as myocardial infarction and chronic heart failure. Characterization of 30-day readmitted patients and risk factors may provide the needed framework for lowering the rate of preventable readmissions after stroke.

## Materials and Methods

This was a retrospective case–control study to identify risk factors predicting 30-day readmission after stroke. A descriptive sub-study of the subset of patients who were readmitted was also conducted as was a mortality analysis of index and readmissions. This study was carried out in accordance with the Hartford Hospital Institutional Revenue Board after the protocol was reviewed and approved.

### Sampling

The study was conducted at a large tertiary care hospital and comprehensive stroke center with a dedicated neurology inpatient service. We identified patients 18 years and older readmitted within 30 days of an index hospitalization between January 1, 2013 and December 31, 2014 with a discharge diagnosis of acute ischemic stroke, hemorrhagic stroke, or transient ischemic attack. Patients readmitted for elective surgeries or palliative care including hospice were excluded from the study. A control group was composed of all other patients, 18 years and older with index admission for stroke during the same time period. Patients who died or were discharged to hospice during the index admission were excluded from the risk analysis as they did not have the same opportunities for readmission; they were included only for analyses on index admission mortality. A total of 134 cases, 1,418 controls, and 248 additional expired patients were identified.

### Data Collection

The majority of data were extracted from a retrospective search of an IRB approved, prospectively maintained data registry (Hartford Hospital Stroke Center Registry) which includes demographic, disease, and treatment variables. Any additional data, not available in the registry, were obtained from the patient electronic medical records.

### Statistical Analysis

For the risk analysis, demographic and other patient characteristics, stroke characteristics, and treatment and other process and timing of care variables were compared between the readmission and control group patients. Treatment by the “neurology service” was defined as patients admitted to the inpatient neurology team as the primary treating service. The overwhelming majority of these patients are admitted to the dedicated stroke unit with specialized nursing care. A minority of patients admitted to the neurology service but not in the stroke unit (due to overflow) were also included and designated as “non-neurology floor.” Categorical variables were assessed using chi-square tests of proportion. Continuous variables, such as age or BMI, were compared using independent group *t*-tests if they met distribution assumptions of normality in preliminary analysis. If the variables did not meet distribution assumptions, Wilcoxon ranked sum test was alternatively used, or the variables were dichotomized and chi-square tests were applied.

Variables identified as likely risk factors (differences between cases and controls with significance values of 0.1 or lower) were then included in a multivariate logistic regression predicting readmission using simultaneous entry process.

For the sub-analyses of patients readmitted within 30 days, the same analytic tests were used in exploring the relationships between index and readmission characteristics and among the readmission characteristics; these included reason for and timing of readmission, disposition after readmission, and (of special focus) risk factors of mortality during the readmission following stroke. All analyses were conducted in SPSS v 21.

## Results

### Risk Factors for Readmission

Several patient characteristics were significantly related to the risk of readmission. Patients over the age of 75 (*p* = 0.02) and those living in a facility prior to index stroke (*p* = 0.01) were significantly more likely to be readmitted. Those living at home without a spouse were also more likely to be readmitted but the association was not significant (*p* = 0.06). Comorbidities, including prior stroke (*p* = 0.03), atrial fibrillation (*p* = 0.03), chronic heart failure (*p* ≤ 0.01), or diabetes mellitus (*p* = 0.03), were also significant risk factors. Among process variables, admission to non-neurology service or non-neurology floor during index admission were also significant (*p* ≤ 0.01; 0.02, respectively). Those not discharged home or discharged with higher NIHSS score (greater than 5) on index admission were also more likely to be readmitted (*p* ≤ 0.01) (Table 1).

**Table 1:** Risk factors for stroke 30-day readmission.

Risk factors	Among controls	Among cases	*p*-Value	OR in multivariate LR (*n* = 1,085)	*p*-Value (LR)

	*N* = 1,418	*N* = 134			
**At admission for index stroke**					
Male gender	699 (49.3%)	67 (50.0%)	0.88		
Age >75	555 (39.1%)	67 (50.4%)	0.02	1.18 (0.77–1.81)	0.456
Living without spouse	639 (45.1%)	49 (36.6%)	0.06	1.26 (0.84–1.88)	0.796
Residence in facility	94 (6.7%)	17 (13.1%)	0.01	1.41 (0.75–2.68)	0.283
Obese (BMI > 30)	452 (34.3%)	39 (31.0%)	0.45		
Prior stroke	297 (20.9%)	39 (29.1%)	0.03	1.39 (0.91–2.12)	0.129
Diabetes mellitus	473 (33.4%)	57 (42.5%)	0.03	1.26 (0.85–1.87)	0.250
Chronic heart failure	156 (11.0%)	29 (21.6%)	<0.01	1.63 (0.99–2.67)	0.288
Atrial fibrillation	282 (19.9%)	38 (28.4%)	0.03	1.26 (0.80–1.99)	0.328
High cholesterol	851 (60.0%)	79 (59.0%)	0.81		
Hypertension	1,138 (80.3%)	109 (81.3%)	0.76		
Depression	277 (19.5%)	28 (20.9%)	0.70		
Dementia	138 (9.7%)	20 (14.9%)	0.06	1.13 (0.63–2.03)	0.684
Hemorrhagic stroke	314 (22.3%)	25 (18.7%)	0.59		
Ischemic stroke	940 (66.6%)	92 (68.7%)			
TIA	157 (11.1%)	17 (12.7%)			
Admit to non-neurology service	195 (15.6%)	40 (32.0%)	<0.01	2.04 (1.28–3.27)	0.003
On non-neurology floor	555 (39.7%)	67 (50.8%)	0.02	1.10 (0.72–1.68)	0.440
**Adding index admission outcomes**					
D/C other than home	695 (49.4%)	84 (63.6%)	<0.01	1.43 (0.75–2.76)	0.577
D/C NIHSS ≥6	205 (24.2%)	30 (42.3%)	<0.01	2.37 (1.21–4.65)	0.012

### Readmissions

**Figure 1:**
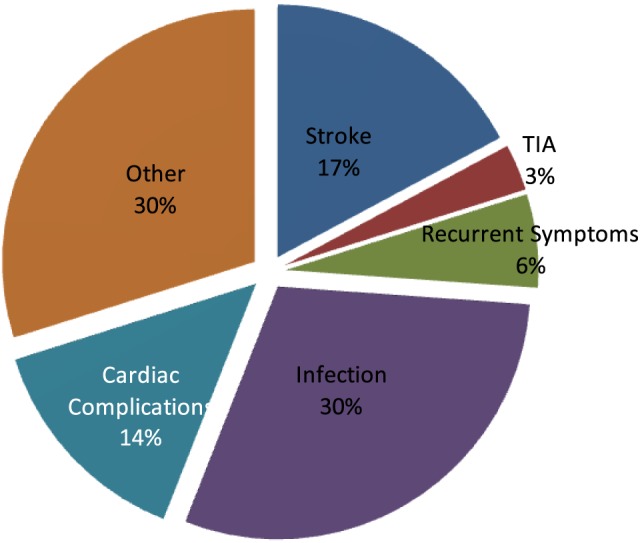
Reasons for readmission.

The stroke 30-day readmission rate for Hartford Hospital during the study’s 2-year time frame was 8.7%. The most common reason for readmission was infection (30%), mostly urinary or respiratory. Other readmission reasons included recurrent stroke or TIA (20%), and cardiac complications (14%). Recurrent symptoms of the initial stroke accounted for another 6%. The remaining 30% of readmission diagnosis were many and included a multitude of etiologies (seizures, falls, and non-infectious respiratory, gastrointestinal, renal, hematologic and orthopedic complications); however, no single etiology reached significant numbers to categorize for analysis (Figure 1).

Patients suffering a recurrent stroke or TIA were more likely to be admitted within 1 week of index discharge (*p* = 0.039). Patients readmitted for recurrent stroke or TIA-related diagnoses were younger, more likely to live at home, and were more likely to have had before stroke. The readmitted patients were less likely to have comorbidities of COPD, coronary heart disease, chronic kidney disease, or have had a recent (pre-index visit) infection than those patients readmitted for other reasons. During the index visit, the patients readmitted for stroke or TIA had significantly shorter index lengths of stay, were more likely to have been discharged home, and were less likely to have been treated with a Foley catheter or PEG (the later showing a trend but not significant) (Table 2).

**Table 2:** Readmission and index visit characteristics for new stroke event (stroke or TIA) vs. others.

	Reason for readmission		
	New stroke or TIA (*n* = 27)	Other reason (*n* = 107)	*p*
**Readmission characteristic**			
Days to readmit (median, IQR)	5 (2, 10)	8 (4, 14)	0.059
Readmit within 1 week (*n*, %)	16 (69.2%)	49 (46.7%)	0.039
Readmission LOS—all (median, IQR)	4 (2, 7)	4 (3, 8)	0.462
Readmission LOS—d/c alive only (median, IQR)	4 (2, 7)	4 (2.75, 8.25)	0.571
**Index visit characteristic**			
Age (median, IQR)	66 (53, 80)	76 (61, 86)	0.105
Pre-stroke live at home (*n*, %)	26 (96.3%)	89 (83.2%)	0.121
Comorbidities (*n*, %)			
Prior stroke	13 (48.1%)	30 (28.0%)	0.045
COPD	1 (3.7%)	18 (16.8%)	0.121
Recent infection	1 (3.7%)	24 (22.4%)	0.026
CAD	3 (11.1%)	39 (35.5%)	0.014
Chronic kidney disease	1 (3.7%)	27 (25.2%)	0.014
Hemorrhagic	8 (29.6%)	17 (15.9%)	0.048
Ischemic	13 (48.1%)	78 (72.9%)	
TIA (*n*, %)	6 (22.2%)	12 (11.2%)	
LOS (median, IQR)	3 (2, 7)	5 (3, 10)	0.027
**Index D/C other than home**	9 (34.6%)	75 (70.8%)	0.001
Treatment with Foley catheter	7 (25.9%)	59 (55.1%)	0.007
Use of PEG	1 (3.7%)	19 (17.8%)	0.076

### Index and Readmission Mortality

Following 30-day readmission after stroke or TIA, patients were more than three times more likely to expire than patients on index admission [36.6 vs. 13.8%, *p* < 0.001; OR = 3.6 (96% CI = 2.5–5.3)]. Patients readmitted for recurrent TIA or stroke were significantly more likely to expire during readmission (*p* = 0.006). When the readmission occurred or how long the readmission lasted was not associated with mortality. During index admission, type of stroke (hemorrhagic) and admission to non-neurological service were significantly related to increased mortality. Prior stroke was not related on index admission (Table 3).

**Table 3:** Mortality—index and readmission visits.

	Mortality		
	D/C alive	Expired or D/C to hospice	*p*
wIndex admission (*n*, %)	1,552 (86.2%)	248 (13.8%)	<0.001
Readmission	85 (63.4%)	49 (36.6%)	OR = 3.6 (2.5–5.3)
Readmission visit mortality	1,552 (86.2%)	248 (13.8%)	
Readmission dx (*n*, %)			
Recurrent stroke	10 (43.5%)	13 (56.5)	*
TIA	1 (25.0%)	3 (75.0%)	
Recurrent stroke symptoms	6 (75.0%)	2 (25.0%)	
Infection	29 (72.5%)	11 (27.5%)	
Cardiac complications	12 (63.2%)	7 (36.8%)	
Other	27 (67.5%)	13 (32.5%)	
Days to readmit (median, IQR)	8 (3, 13.5)	6 (3, 15.5)	0.482
Index LOS (median, IQR)	5 (3, 9)	5 (3, 9)	0.886
Index visit mortality	1,552 (86.2%)	248 (13.8%)	
Prior stroke	336 (88.2%)	45 (11.8%)	0.210
Admission non-neurology service	2,235 (77.8%)	67 (22.2%)	<0.001
Type of stroke			<0.001
Hemorrhagic	365 (75.5%)	110 (24.5%)	
Ischemic	1,032 (88.2%)	138 (11.8%)	
TIA	174 (100.0%)	0 (0.0%)	<0.001

*Statistics for new stroke or TIA vs. all others: *p* = 0.006; OR = 3.37 (1.4–7.8).

## Discussion

Although readmission is common after stroke, there remains a lack of consistent understanding on this matter due to a multitude of variables. Moreover, there is paucity in the literature in regards to the associated mortality risk with 30-day readmission. In 2006, a nationwide study found that older age, and cardiovascular comorbid conditions were strong indicators of preventable readmissions (6). Furthermore, a statistical significant association was found between elderly patients, premorbid functional status, and readmissions (7). Our data confirm both of these findings. In a comprehensive study with 253,680 patients, the three major causes of 30-day readmissions were infection, CAD, and recurrent stroke (8). Approximately one-third of 30-day readmissions were infection related and one in five patients returned with a recurrent stroke or TIA, comparable to previously listed studies. The overwhelming majority of infections in our study were urinary tract related (47.5%) and respiratory (42.5%). Both have commonly observed with stroke patients due to recumbence, indwelling urinary catheters, and aspiration risk. Patients with higher risk for infection may benefit from preventative interventions, sooner outpatient follow-up, and early treatment.

Other studies, focused on hemorrhagic stroke, found that infection was a common cause for 30-day readmission (9). Recent studies have also found that inpatient procedures and complications lead to an increase in readmission rates (5). Although studies have been successful in characterizing stroke 30-day readmission, consistent and thorough data are needed.

Our study design comprised analysis of several factors and determinants of readmissions. Patient characteristics, social circumstances, clinical processes of care, and health outcomes were all considered. The mortality analysis of 30-day readmissions study was strong as index mortality was considered, and hospice discharge designations were excluded. Index admission to non-neurology service was an independent risk factor of 30-day readmissions. The overall mortality associated with stroke 30-day readmission was high. Patients treated at specialized centers in the context of stroke systems of care have better outcomes and lower complication rates. For example, a Cochrane review encompassing 5,855 stroke patients compared dedicated hospital stroke unit care vs. non-dedicated or general care. Patients who receive organized inpatient care in a stroke unit were more likely to survive and be independent and living at home 1 year after the stroke irrespective of age, sex, stroke severity, subtype, or increased length of stay (10).

Factors associated with stroke readmission have been characterized in five domains: patient characteristics, social circumstances, clinical processes of care, health outcomes, and health system determinants (including hospital location and treating physician) (11). Although treating physician (neurology vs. non-neurology service) was investigated in our analysis, location of the hospital proximity of patients (12) and insurance type (13, 14) were not analyzed and have been previously demonstrated to influence readmission.

Other limitations were presented due to the variables themselves. For example, many large tertiary care hospitals and academic comprehensive stroke centers like our institution receive patient transfers form other hospitals. The index admission variable of whether patients were transferred is an interesting aspect to consider and proved to be significant in relation to readmission. Within 30 days, patients are more likely return to their original hospital for reasons such as infection or other complications from their initial stroke. Although the 30-day stroke readmission timeline has been proposed as a measure to judge the level of patient care and hospital competence, this and other studies confirm the potential downfalls and limitations of this metric are abundant (5).

## Conclusion

Mortality among stroke readmission is high and numerous factors influence this metric. As many preventable and/or devastating readmissions occur within the first 3 weeks of discharge, early follow-up for high risk patients may be beneficial to prevent readmission and the associated mortality risks. With these findings, it becomes clear that 30-day readmissions in stroke patients are an occurrence worth consideration and analysis in designated stroke intuitions to tailor plans pertaining to their treated population.

## Author Contributions

AN designed the study and originated the hypothesis. IS staff performed the statistical analysis and aided in study methodology. All authors contributed to data extraction, review, and analysis. The manuscript design was completed by LM and AN.

## Conflict of Interest Statement

The authors declare that the research was conducted in the absence of any commercial or financial relationships that could be construed as a potential conflict of interest.
